# The impact of olfactory dysfunction on interoceptive awareness

**DOI:** 10.1111/psyp.12316

**Published:** 2014-08-11

**Authors:** JACQUELINE KRAJNIK, KATHRIN KOLLNDORFER, LISBETH A. NOTTER, CHRISTIAN A. MUELLER, VERONIKA SCHÖPF

**Affiliations:** a Department of Biomedical Imaging and Image-guided Therapy, Medical University of Vienna, Vienna, Austria; b Department of Otorhinolaryngology, Medical University of Vienna, Vienna, Austria

**Keywords:** Anosmia, Heartbeat perception, Hyposmia, Interoceptive awareness, Olfactory dysfunction, Smell

## Abstract

This study aimed to investigate how the impairment of the olfactory system influences interoception. Interoception is known as the awareness of one’s body or the sense of the condition of the body; more precisely, this construct is defined as the processing, representation, and perception of the internal physical state. Interoceptive sensitivity and chemosensory performance were assessed in 77 subjects, including 43 functional anosmics, 18 hyposmics, and 16 healthy controls. Interoceptive awareness was predicted by odor detection threshold, as well as the duration of olfactory loss in patients who suffered from reduced olfactory function—the longer the olfactory impairment, the worse the perception of bodily signals. The results of this study will significantly contribute to the basic understanding of the multifaceted effects of olfactory alterations.

Among the general population, the prevalence of olfactory impairment seems to increase with age: population-based studies found a prevalence of 19% for olfactory dysfunction (5% for anosmia, the total loss of the sense of smell, and 15% for hyposmia, which is classified as reduced olfactory sensitivity) in individuals over 20 years of age (Brämerson, Johansson, Ek, Nordin, & Bende, 2004), and a rate of approximately 22% for individuals between 25 and 75 years of age (Vennemann, Hummel, & Berger, 2008). Another survey found a prevalence rate of 24% for persons over 53 years of age (Murphy et al., 2002). The causes of olfactory dysfunction are multifaceted, but the most common ones are head trauma, infections of the upper respiratory tract, sinunasal diseases (e.g., nasal polyps), and smell disorders related to neurodegenerative disorders (Hüttenbrink, Hummel, Berg, Gasser, & Hähner, 2013; Keller & Malaspina, 2013), such as multiple sclerosis (Rolet et al., 2013) and Parkinson’s disease (for a review, see Doty 2012).

The effects of olfactory dysfunction can be multilayered: several studies have demonstrated that the quality of life in individuals who suffer from reduced olfactory perception is negatively impaired (for a review, see Croy et al., 2014). These patients complain about the inability to identify dangerous situations, like the perception of gas, fire, or smoke, or to notice spoiled food (Hummel & Nordin, 2005; Keller & Malaspina, 2013; Miwa, 2001; Nordin, Blomqvist, Olsson, Stjärne, & Ehnhage, 2011; Stevenson, 2010). Furthermore, interpersonal relationships are highly influenced by the inability to perceive body odors, since this lack leads to tremendous uncertainty (Hüttenbrink et al., 2013). Based on these conditions, it is accordingly not surprising that olfactory disorders often result in impairment of emotional well-being. Studies by Pollatos et al. (2007), as well as Croy and colleagues (2014), have reported that reduced olfactory perception was also associated with depressive symptoms. In addition to its impact on quality of life and emotional well-being, reduced olfactory function has also been associated with altered body weight and body mass index (BMI, Aschenbrenner, Hummel et al., 2008). Apart from olfactory dysfunction, interoceptive awareness interrelates with depressive symptoms, more precisely, with psychiatric disorders characterized by emotional impairment (Furman, Waugh, Bhattacharjee, Thompson, & Gotlib, 2013; Pollatos, Traut-Mattausch, & Schandry, 2009; Stevens et al., 2011). Dietary changes were reported by patients with a loss of the sense of smell, such as eating less since the onset of the disease, or alterations in food preferences, such as eating spicier foods (Aschenbrenner, Hummel et al., 2008). Studies that have focused on specific diseases that correlate with the BMI found poorer olfactory performance (Aschenbrenner, Scholze, Joraschky, & Hummel, 2008), as well as decreased olfactory awareness for food stimuli (Schreder et al., 2008) in anorectic patients. In addition to reduced olfactory function, anorectic as well as bulimic patients were found to show deficits in interoceptive awareness (Klabunde, Acheson, Boutelle, Matthews, & Kaye, 2013; Pollatos et al., 2008).

Interoception refers to the processing, representation, and perception of the internal physical state. More precisely, this construct involves the sensation of the physiological condition of the body, as well as the representation of the interior state within the context of ongoing actions, and quantifies the extent of an individual′s sensitivity to these stimuli that originate from within the body (Ainley & Tsakiris, 2013; Cameron, 2001). Interoceptive body responses can be measured and obtained using heart rate, respiration, and blood pressure (Cameron, 2001). Further, both olfactory dysfunction (Aschenbrenner, Hummel et al., 2008) and interoceptive awareness (Klabunde et al., 2013; Pollatos et al., 2008) are linked to altered body weight and BMI, respectively, in disorders characterized by altered body weight.

Based on the literature, the question of a potential relationship between olfactory dysfunction and interoception emerges. Furthermore, as body perception is linked to the perception of the environment, the following question also arises: How does the loss or impairment of a specific sense (olfaction) affect the awareness of one’s own body? Consequently, the primary aim of our study was to assess the impact of olfactory dysfunction on the accuracy of interoceptive awareness. Due to the suggested influence of olfactory dysfunction on depressive symptoms and BMI, both variables were analyzed in the study design.

## Method

The study protocol was evaluated and approved by the local ethics committee of the Medical University of Vienna, Austria, according to the Declaration of Helsinki.

### Subjects

The study was designed as a retrospective study investigating interoceptive sensitivity on a selected group (olfactory dysfunction) of a larger study population that was acquired in a different context, but including the questionnaires and tasks necessary for the current investigations. Of the 80 subjects initially included in the study, three had to be excluded due to incomplete measurements. Therefore, data from 77 subjects (32 males, 45 females) between the ages of 18–73 years (mean = 46.6; *SD* = 11.3) at the time of measurement were included. None of the subjects had a history of any neurological or psychiatric disease. After olfactory testing (see Procedure and Materials), they were classified according to olfactory performance, resulting in 43 anosmics (18 males, 25 females), 18 hyposmics (7 males, 11 females), and 16 healthy controls (7 males, 9 females). None of the participants reported taking any kind of medication at the time of testing. For further sample description see Table 1.

### Procedure and Materials

All participants were recruited either by newspaper advertisements or announcements at local ear, nose, and throat (ENT) physicians and clinics, or were referred to our study by the ENT Department of the Medical University of Vienna. A detailed patient history was acquired, and patients who matched the following criteria were included: no history of neurological, psychiatric, or psychosomatic diseases; and no use of medication and presence of an olfactory dysfunction (TDI score < 31, see next paragraph). Moreover, a full entorhinal examination by an ENT physician (C.A.M.) was offered.

All included participants were tested individually following the same test protocol. Participants were informed about the study, gave their written informed consent, and sociodemographic details were collected. In addition to acquiring general information about age and sex, this questionnaire addressed important criteria, such as history of neurological, psychiatric, or psychosomatic diseases, or use of medication. Subsequently, olfactory performance was tested using the Sniffin’ Sticks battery (Burghart Instruments, Wedel, Germany), consisting of three subtests—detection threshold, odor discrimination, and odor identification—all assessing different dimensions of olfactory function (for a detailed description of the test procedure, please see, e.g., Hummel, Sekinger, Wolf, Pauli, & Kobal, 1997; Kobal et al., 1996). Combining the outcomes of all three subtests, the so-called TDI score was built. The TDI score gives normative information on olfactory function used for clinical diagnosis. Normosmics, that is, subjects with normal chemosensory function, are defined by a TDI score of at least 31. Hyposmia (reduced olfactory function) is defined as a TDI between 17 and 30.75, and a score of less than 17 is categorized as anosmia (Kobal et al., 2000).

In order to assess the accuracy in detecting bodily signals, the heartbeat perception task (HBPT), known as the gold standard in detecting interoceptive deficits, was performed by using a modified version of the heartbeat perception task of Schandry (1981). Subjects were instructed to close their eyes, relax, and think of special situations, including physically demanding activities, that is, activities where the heartbeat is typically more intense. Subsequently, they were asked to count their own heartbeats silently during three varying heartbeat-counting phases (30 s, 20 s, 40 s), which were indicated by a start and stop signal from the investigator. Simultaneously, the investigator was counting the participant’s heartbeats by taking their pulse at the wrist. After each counting phase, participants were encouraged to verbally report the number of perceived heartbeats. As a direct consequence of this procedure, subjective and objective measurements of heartbeats were obtained. Due to the known impact of olfactory impairment on the BMI and depressive symptoms (for a review, see Croy, Nordin, & Hummel, 2014), subjects also underwent a questionnaire designed to reveal depressive symptoms (Beck Depression Inventory-II, BDI-II; Beck et al., 1996). The BMI was evaluated using the body weight and body height of subjects, ascertained by the sociodemographic questionnaire. The whole study protocol lasted approximately 1 1/2 h.

### Statistical Analysis

To directly evaluate the impact of olfactory dysfunction on the collected measures, subjects were clustered into two groups: normosmic participants (norm), and subjects who suffered from olfactory dysfunction (olf dys) for all analyses. Interoceptive awareness was evaluated by computing a heartbeat perception index based on the following formula: 1/3∑ (1 – (∣recorded-counted∣)/recorded). Corresponding to this transformation, the mean score of the three counting phases was calculated. The resulting scores could vary between 0 and 1, in which higher scores would indicate a smaller disparity between the subjective (heartbeats counted by participant) and objective (heartbeats recorded by investigator) heartbeats.

Statistical analyses were performed using the Statistical Package for the Social Sciences, Version 20.0 (SPSS, Chicago, IL). Multiple regression analyses were performed to evaluate potential variables that could predict interoceptive awareness for both cohorts. To analyze the impact of olfactory impairment on the BMI and depressive symptoms (BDI), nonparametric methods were used, since the assumption of normal distribution was not fulfilled. The alpha level for all tests was set at .05 (two-tailed).

## Results

As the main focus of this study was to assess whether interoception is impaired in patients with olfactory dysfunction, compared to normosmic subjects, the cohort was clustered into two groups: normosmic participants (norm) and subjects who suffered from olfactory dysfunction (olf dys). Statistical testing was consequently evaluated with respect to these groups.

Using multiple regression analyses, potential predictive variables for interoceptive awareness were evaluated for both groups separately. Using stepwise iterations, the following predictor variables were included in the model: TDI score; age; BMI; BDI scores; olfactory threshold score; and, for regression analysis of the olfactory dysfunction group, the duration of the dysfunction.

**Figure 1:**
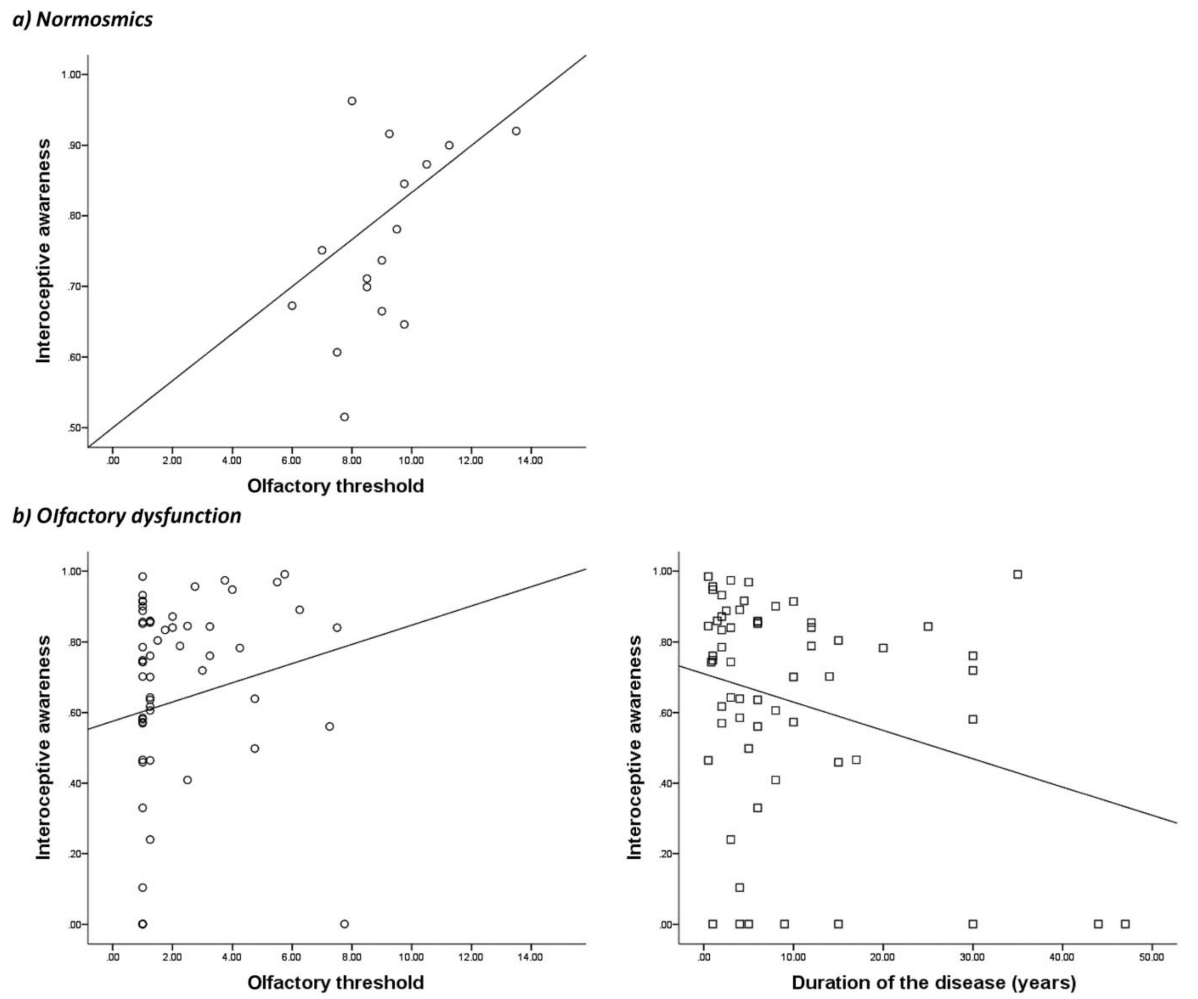
Multiple regression analysis revealed the olfactory threshold as a significant predictive variable for interoceptive awareness in normosmic patients, explaining 25.4% of the variance (a). In addition, the same method for the olfactory dysfunction group obtained two predictive factors for interoception, olfactory threshold and duration of the disease, with 24.5% of the variance explained (b).

Results of the regression for normosmic participants (16 subjects) indicated the predictor “olfactory threshold score” to explain 25.4% of the variance (*R*^2^ = .30, *F*(1,15) = 6.120, *p* = .027), thus the predictor significantly predicted interoceptive awareness (β = .552, *p* = .027; Figure 1a).

Results of regression analysis for the olfactory dysfunction group (including 47 complete data sets of participants) indicated two predictors to explain 24.5% of the variance (*R*^2^ = .28, *F*(2,46) = 8.484, *p* = .001). The significantly tested model to explain interoceptive awareness in patients with olfactory dysfunction included disease duration (β = −.405, *p* = .003) and the olfactory threshold score (β = .402, *p* = .003; Figure 1b).

**Figure 2:**
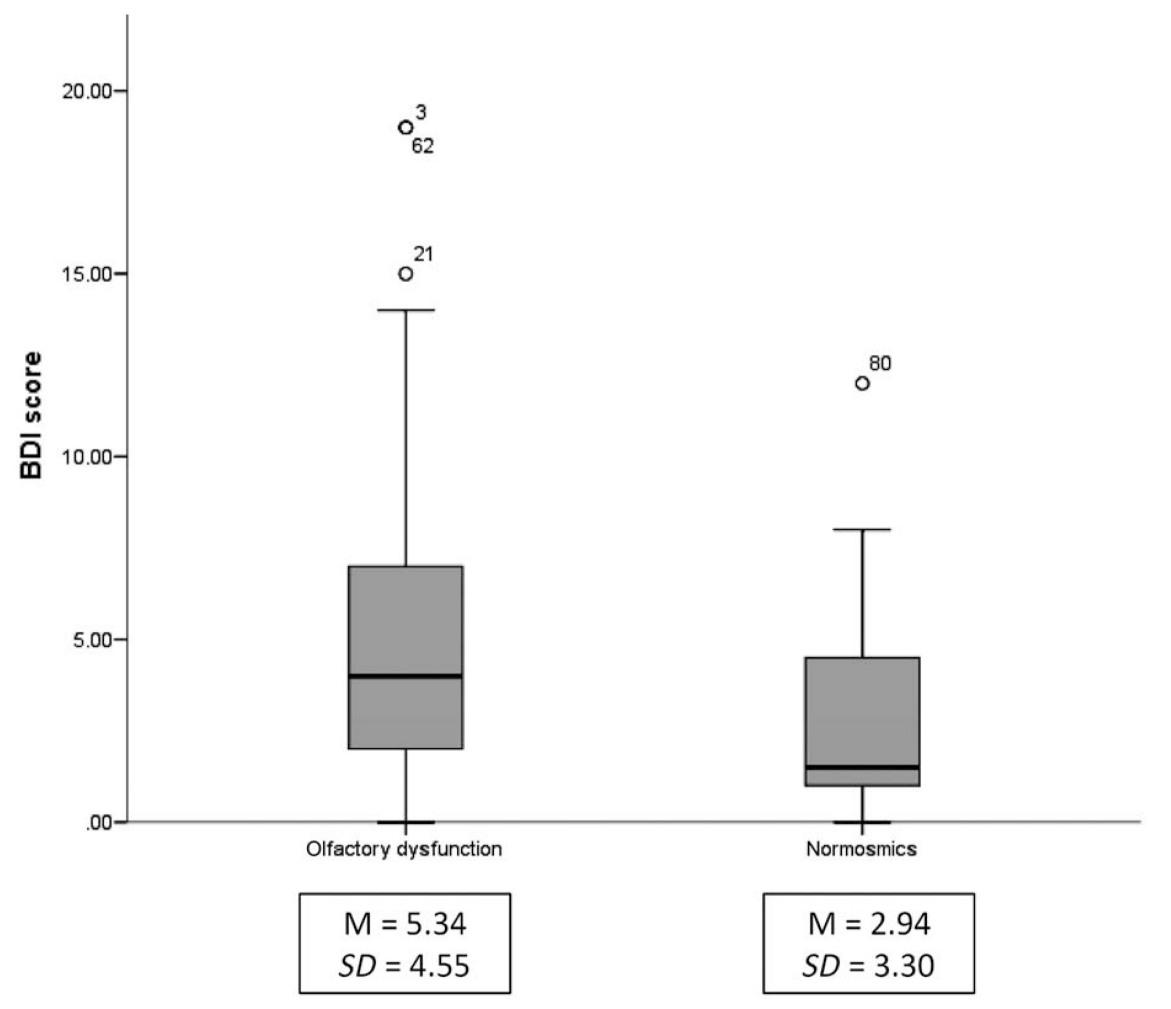
Box plot diagram depicting data distribution of BDI scores of the two groups, olfactory dysfunction and normosmics. Due to incomplete BDI data of the olfactory dysfunction group, 53 patients (41 anosmics, 12 hyposmics) were compared to 16 healthy controls. Associated mean BDI scores (*M*) and standard deviation scores (*SD*) are represented below each group.

With regard to the known impact of olfactory impairment on the BMI and depressive symptom scores obtained by BDI, Mann-Whitney tests were executed for both groups. While BMI scores tended to differ between normosmics (median = 22.9) and subjects with reduced olfactory function (median = 25.0) *U* = 390, *z* = −1.93, *p* = .054, a significant difference between the groups (norm: median = 1.5; olf dys: median = 4.0) for depressive symptoms, *U* = 268, *z* = −2.23, *p* = .026 (see Figure 2) was observed.

## Discussion

The aim of our study was to investigate whether a relationship between olfactory dysfunction and interoceptive awareness exists, in order to obtain more insight into how impairments of olfactosensory input may affect the representation of the internal physical state. Investigations in 77 subjects classified according to olfactory performance (two groups: normosmics and participants who suffered from olfactory dysfunction) revealed the olfactory threshold as a significant predictor of interoceptive awareness in both normosmics and subjects who suffered from olfactory dysfunction, with higher olfactory threshold scores indicating better interoceptive awareness. This result is not surprising, considering that the olfactory threshold score represents a powerful clinical parameter; in addition, both olfactory threshold and interoceptive awareness represent various forms of sensitivity. For subjects who suffered from olfactory dysfunction, the multiple regression model further revealed that a combination of duration of disease and olfactory threshold values could explain interoception awareness measures. The longer patients suffered from reduced olfactory function, the worse the ability to perceive bodily signals.

Several studies have shown that olfactory training can increase recovery rates in patients with postinfectious and posttraumatic olfactory dysfunction (Damm et al., 2013; Konstantinidis, Tsakiropoulou, Bekiaridou, Kazantzidou, & Constantinidis, 2013); thus, olfactory training could be a promising therapeutic approach to improve olfactory skills. Based on this and our main findings, two questions arise that should be part of future investigations: How will interoceptive awareness be altered if olfactory function is regained through olfactory training? And, second, due to the unknown causality (cause-effect relation) between olfactory dysfunction and interoceptive awareness, is it possible to positively affect interoceptive awareness, for example, by mindfulness training (Farb, Segal, & Anderson, 2013) and, consequently, improve the sense of smell?

Previous research has already demonstrated a significant relationship between olfactory perception and eating disorders (Aschenbrenner, Scholze et al., 2008; Dazzi, Nitto, Zambetti, Loriedo, & Ciofalo, 2013; Rapps et al., 2010; Schreder et al., 2008), and between eating disorders and interoceptive awareness (Klabunde et al., 2013; Pollatos et al., 2008). The results of studies examining olfactory function in patients suffering from anorexia and bulimia nervosa diverge with regard to alterations in olfactory domains (overall TDI, olfactory threshold, identification, discrimination). A recent study confirmed reduced overall olfactory performance in anorectic patients; in addition, patients who suffered from bulimia showed impaired olfactory performance. Both groups showed a lower capacity for discrimination of olfactory stimuli; however, olfactory threshold was altered only in bulimic participants (Dazzi et al., 2013). In addition to reduced olfactory function, anorectic, as well as bulimic patients, were found to show deficits in the perception of bodily signals (interoceptive awareness, Klabunde et al., 2013; Pollatos et al., 2008). In the present study, patients who suffered from olfactory dysfunction tended to display reduced interoceptive awareness and a higher BMI—suggesting a potential relationship between interoception, olfaction, and body weight regulation. Results further showed higher BMI scores for participants who suffered from an olfactory dysfunction than for subjects with no olfactory impairment. Nevertheless, we are aware that the BMI does not serve as a full clinical criterion for the investigation of eating behavior as does, for example, the Eating Disorder Inventory-2 (EDI-2; Garner, 1991), but this result may be a first attempt to motivate future studies addressing the mutual relationship between eating disorders and olfactory dysfunction and interoceptive awareness. Eating disorders are not only accompanied by olfactory dysfunction, but are primarily characterized by reduced interoceptive awareness (Klabunde et al., 2013; Pollatos et al., 2008). Similar to the role of olfactory perception in eating disorders, this causality (cause-effect relationship) is still unresolved. As suggested by Klabunde et al. (2013), it is still not clear whether interoceptive deficits are present prior to the onset of the disease, or whether decreased interoception is a consequence of these severe disorders. Based on the presented results of previous studies and combined with the trends revealed in the current study, further investigation on the interactive relationship between olfactory dysfunction and interoception and eating disorders would be of major interest.

Due to the known impact of olfactory dysfunction on emotional well-being, depressive symptoms were also analyzed in the study design. According to previous findings (Croy, Symmank et al., 2014; Pollatos et al., 2007), significant differences were revealed concerning emotional well-being between the two groups investigated in this study: subjects with olfactory dysfunction had higher mean BDI scores, in general. Nevertheless, it should be stressed, at this point, that the mean BDI scores of normosmic and olfactory dysfunction patients were within the normal range, indicating no current depression (Beck, Steer, & Brown, 1996).

### Limitations of Present Study

A limiting factor of the present study is the size of the compared samples of normosmics and participants who suffered from olfactory dysfunction, which was not optimal, since the control group consisted of 16 subjects. As the focus of our study was to obtain initial insight into the interoceptive awareness of patients with olfactory dysfunction, two groups—patients with olfactory dysfunction and healthy controls—were contrasted, and multiple regression analysis was performed separately for both groups. Although the results of the current study significantly contribute to our knowledge about the multifaceted effects of olfactory dysfunction, larger cohorts including different causes of anosmia would be of interest for further research.

### Conclusion

Overall, our study was the first to examine the effect of olfactory dysfunction on interoceptive awareness, and provides significant insights into the multifaceted effects of olfactory dysfunction. These significant findings suggest a relationship between olfactory dysfunction and interoceptive awareness, and revealed a better perception of bodily signals in healthy controls than in patients who suffered from olfactory dysfunction. They give rise to a new component related to olfactory dysfunction, thus contributing to the multifaceted effects of olfactory alterations. Despite the deteriorated accuracy in detecting bodily signals, participants with reduced olfactory dysfunction showed a trend toward higher BMIs than normosmics. Although the BMI does not provide a complete clinical criterion with which to examine eating behavior, our results may be an initial approach to motivate future studies that will address the mutual relationship between specific factors, eating disorders, olfactory dysfunction, and interoceptive awareness.

## Figures and Tables

**Table 1:** Sociodemographic Data Comparing Normosmic Participants with those Suffering from Olfactory Dysfunction

	Normosmics (*n* = 16)	Olfactory dysfunction (*n* = 61)	Anosmics (*n* = 43)	Hyposmics (*n* = 18)
Cause of olfactory loss		Idiopathic (*n* = 39)	Idiopathic (n = 29)	Idiopathic (*n* = 10)
		Postinfectious (*n* = 18)	Postinfectious (*n* = 10)	Postinfectious (*n* = 8)
		Posttraumatic (*n* = 2)	Posttraumatic (*n* = 2)	
		Polyps (*n* = 2)	Polyps (*n* = 2)	
Age (range) in years	30.6 (18–45)	54.7 (22–73)	54.8 (22–73)	54.6 (24–68)
Gender (m/f)	7/9	25/36	18/25	7/11
Average disease duration (range) in years		9.5 (0.5–47)	9.0 (0.5–44)	10.6 (0.5–47)
Average interoceptive awareness score	0.76	0.63	0.64	0.61
Average body mass index (range)	23.29 (18.42–29.98)	25.86 (17.93–41.73)	26.22 (19.96–41.73)	23.81 (17.93–28.38)
Average TDI score (range)	35.80 (32.5–40.5)	15.67 (7–30.50)	12.20 (7–17)	23.94 (17.25–30.50)
Average threshold score (range)	9.05 (6–13.5)	2.13 (1–7.75)	1.42 (1–4.75)	3.83 (1–7.75)
Average discrimination score (range)	12.94 (10–16)	6.74 (3–13)	5.55 (3–12)	9.55 (6–13)
Average identification score (range)	13.81 (10–15)	6.80 (2–15)	5.23 (2–11)	10.55 (4–15)

*Note*. Although two groups (normosmics and olfactory dysfunction) were included in the statistical data analysis, a more detailed description of the olfactory dysfunction group is given by further splitting of the data into anosmics and hyposmics.
